# Are Men Being Left Behind (Or Catching Up)? Differences in HPV Awareness, Knowledge, and Attitudes Between Diverse College Men and Women

**DOI:** 10.1177/1557988319883776

**Published:** 2019-12-01

**Authors:** Sharice M. Preston, William W. Darrow

**Affiliations:** 1 Department of Health Promotion and Behavioral Sciences, University of Texas Health Science Center School of Public Health, Houston, TX, USA; 2 Department of Health Promotion and Disease Prevention, Robert Stempel College of Public Health and Social Work, Florida International University, Miami, FL, USA

**Keywords:** HPV, college students, HPV vaccine, awareness, knowledge, attitudes

## Abstract

The aim of this cross-sectional survey was to assess awareness, knowledge, and attitudes in regard to human papillomavirus (HPV) and vaccination against HPV among college students. From 2015 to 2017, 386 diverse undergraduates were recruited from a south Florida university. A survey, part of which was researcher developed, of HPV awareness, knowledge, and attitudes was conducted. The majority (84%) of participants had heard of HPV, and 70% had favorable attitudes toward vaccination. Only 28% of men and 55% of women had received ≥1 dose vaccine (*p* = .01), and 4% of all participants reported that they had received 3 doses. Those with ≥1 dose (*n* = 123, 40.1%) were more knowledgeable about HPV (*p =* .01). High knowledge scores were recorded for 30% of respondents and were strongly associated with HPV vaccine initiation among both men and women (*p <* .001) and perceived knowledge among women only (*p <* .001). Negative attitudes toward HPV vaccine acceptance were associated with low knowledge scores (*p* = .01) and undervaccination (*p <* .001). Vaccinated women (*n* = 95) were over seven times more likely than were unvaccinated women (*n* = 115) to report positive vaccine attitudes (relative risk = 7.1). HPV vaccination status was not associated with vaccine attitudes among men. HPV knowledge and vaccine uptake remain problematic among college students, and deficits in both are associated with negative HPV vaccine attitudes. Although the knowledge gap is narrowing, HPV vaccination efforts should target young men, as HPV-related cancer morbidity continues to rise in men.

Human papillomavirus (HPV) infection is the most common sexually transmitted infection (STI) in the world (United States [U.S.] Department of Health and Human Services, 2015), and the HPV vaccine is largely unemployed in the United States as a cancer control resource, especially among men. Although most HPV-infected persons clear the virus completely, HPV can cause genital warts and cancers in both men and women. Due to the long-established connection between some HPV subtypes and cervical cancer, women were initially thought to be the only population burdened by the clinical outcomes of the virus (Gissman et al., 1983; Walboomers et al., 1999), resulting in treatment and prevention solutions that targeted women solely (Celentano, Klassen, Weisman, & Rosenshein, 1988; Jain & Barone, 1996; Slater, Bar-Cohen, Korn, & Yawn, 1994). Several studies estimate that HPV incidence and prevalence in men are similar to those of women, although substantial variability may exist by location and population (Dunne, Nielson, Stone, Markowitz, & Giuliano, 2006).

HPV is the cause of over 90% of genital warts in men, who experience a longer duration of this outcome and incur more treatment costs than women do. HPV also is responsible for the majority of male anal, oropharyngeal, and penile cancers, creating substantial health and economic burden beyond that of cervical cancer (Colón-López, Ortiz, & Palefsky, 2010; Giuliano et al., 2008). The direct medical costs of HPV-related outcomes in the United States were estimated to be $1.7 billion in 2008 (Owusu-Edusei, Chesson, Gift, Tao, & Mahajan, 2008). HPV-related cases of oropharyngeal cancers in otherwise healthy men were predicted to surpass the number of cervical cancer cases in the United States by the year 2020 if HPV vaccination rates are not improved (Chaturvedi et al., 2011). This happened sooner than expected, with new cervical cancer cases dropping each year as oropharyngeal cancers rose, 82% of the latter occurring in men (Van Dyne et al., 2018). Despite this trend, men’s knowledge about HPV has lagged behind women’s.

The Advisory Committee on Immunization Practices (ACIP) has recommended safe and effective HPV vaccination for girls and women aged 9 to 26 years since 2006 (Meites, Kempe, & Markowitz, 2016). Substantial decreases in vaccine-preventable HPV-subtype infections were evident after only the sixth year of vaccine implementation in the United States; a 64% decrease was observed among those 14 to 19 years old and a 34% decrease among those 20 to 24 years old (Markowitz et al., 2016). The quadrivalent HPV vaccine was additionally recommended for boys and young men in 2011 (Meites et al., 2016). However, HPV vaccination rates among boys and men in the United States are far below the recommended Healthy People 2020 target of 80% coverage (U.S. Department of Health and Human Services, 2017). Regionally, vaccination coverage is lowest among males in the Southern United States as compared to other regions (Reagan-Steiner et al., 2016).

HPV vaccine acceptability among men is highly correlated with health-care provider recommendations, although providers more consistently recommend the vaccine for females than for males (Lieblong, Montgomery, Su, & Nakagawa, 2019). Low awareness of HPV vaccination for men is also driven by the absence of HPV screening tests and approved therapeutic treatments for HPV infections in men.

For college students, given this population’s increased sexual activity and sexual risk-taking, as compared to other age groups, HPV infection constitutes a substantial threat (Grello, Welsh, & Harper, 2006; Guttmacher, 1994; Wechsler, Dowdall, Davenport, & Castillo, 1995). College-aged young adults (20 to 24 years old) have the highest prevalence of anogenital HPV infection as compared to other age groups (Giuliano et al., 2008; Markowitz et al., 2016). Most universities provide on-site medical facilities for students, offering HPV vaccination and education among other preventive services (Barnett et al., 2016). HPV-related knowledge in college students is low compared to that of other STIs, especially among men (Fontenot, Fantasia, Charyk, & Sutherland, 2014).

The very-slow-growing HPV vaccination rates leave a large population of college-aged young adults unprotected, despite eligibility to receive catch-up doses up to age 26 years (Mehu-Parant, Rouzier, Soulat, & Parant, 2010). Vaccine acceptability is correlated with awareness and knowledge of HPV (Ferris et al., 2009; Gerend & Barley, 2009; Hunter & Weinstein, 2016; Lenselink et al., 2008); yet, until recently, little research has included college-aged men’s attitudes toward HPV vaccination (Brewer, Ng, McRee, & Reiter, 2010; Fitzgerald, Savage, & Hegarty, 2014). Research focused on characterizing disparities in awareness and knowledge is especially needed in diverse areas, such as the state of Florida, which has some of the lowest HPV vaccination rates in the country as well as some of the highest cervical cancer rates (Fontenot, Fantasia, Sutherland, & Lee-St. John, 2016). An evaluation of what men and women in college know, think, and do about the threat of HPV can offer possible solutions to remedy the underutilization of the HPV vaccine in a population at high risk for contracting this preventable infection. Thus, this research explores differences in HPV-related awareness, knowledge, attitudes, and practices among men and women attending college in south Florida.

## Methods

A cross-sectional study of university students that focused on students’ HPV-related awareness and knowledge, as well as attitudes and uptake of HPV vaccination, was conducted from March 2015 to February 2017. The university’s institutional review board granted exempt approval status prior to study implementation.

### Participants

The male and female students recruited for this study were from one of the largest institutions of higher learning in the United States. Participating students were enrolled in four sections (semesters) of an undergraduate gender studies course taught by the same instructor from March 2015 to February 2017. This course fulfilled a graduation requirement and was typically populated by students from diverse studies. Students enrolled in undergraduate public health and dietetics courses in January and February 2017 also were recruited. Students aged 18 years or older who were present on the day of data collection were invited to participate by completing a questionnaire. Participants were read a brief statement regarding voluntary participation and consent. Students were not required to answer every item or return the questionnaire to the study team. Students were included in the current study if they were (a) 18 years or older at the time of data collection, (b) registered for the college course in which data collection took place, and (c) present in class at the commencement of data collection.

### Survey Procedures

At the time of data collection, the research team explained the purpose of their visit to the class and presented a statement of informed consent. Participants under the age of 18 years were identified and excluded from participation but were welcome to remain in the classroom. Written, self-administered questionnaires were then distributed to eligible participants. The survey took approximately 5 to 10 min to complete. Upon completion, surveys were deposited by each student into a large envelope or box carried by circulating research team members. Participants received tokens of appreciation for their time, such as promotional pens, pencils, lip balm, and condoms.

### Measures

The questionnaire consisted of 34 items. Nine were sociodemographic or descriptive items and 18 items were adapted from the 100-item 2009 University of North Carolina Men’s Health Survey (Brewer et al., 2010; Reiter, Brewer, McRee, Gilbert, & Smith, 2010). The remaining items were developed in March 2015, after a review of the literature, and were based on the aims of the study. Questionnaires were pretested with a focus group of 16 graduate public health students and revised based on feedback (the questionnaire is available from the authors upon request).

Sociodemographic and baseline data (i.e., age, sex, identity, ethnicity/race, health insurance status, sexual orientation, relationship status, sexual activities, and number of partners) were self-reported. Awareness of HPV was assessed with a single question: “Have you heard of HPV or human papillomavirus before today?” (“yes,” “no,” “don’t know”). Participants then indicated how many doses of HPV vaccine they had received as of that day (0, 1, 2, 3, “don’t know”), and, subsequently, if they were not already fully vaccinated, whether they were willing to take the HPV vaccine (“yes,” “no,” “don’t know”). Participants also responded to the statement “Before today, I have never thought of getting the HPV vaccine for myself” (“yes,” “no,” “don’t know”).

Eleven questions concerned beliefs about various HPV-related statements, answered on a 5-point Likert scale that ranged from *strongly agree* to *strongly disagree* with an additional *don’t know* option. An ad hoc scale, consisting of the five Likert items that specifically addressed vaccination, was developed. This scale was used to gauge participants’ attitudes toward the HPV vaccine, whom they saw as appropriate recipients, and vaccines in general (e.g., “Vaccines in general are safe and effective,” “My opinion of the HPV vaccine is mostly positive”). Items were coded such that *strongly agree* or *agree* corresponded to a positive attitude toward that topic. Reliability assessment of the vaccine attitudes scale was conducted with Cronbach’s alpha, with acceptable reliability (*n* = 397, *α* = 0.91).

Participants were then asked to describe their perceived level of knowledge of HPV and the vaccine in answering two separate questions with “Nothing at all,” “a little,” “a moderate amount,” or “quite a lot.” In addition to respondents’ reported self-perceived knowledge related to HPV, knowledge was measured with seven true/false items. A composite knowledge score was created by summing the number of correct responses (maximum of seven; Appendix 1). Respondents’ knowledge was categorized as either high (four or more questions correct) or low (three or fewer questions correct). Participants were not required to answer each question before proceeding to the next and had the option of selecting “don’t know” in the baseline questionnaire. Percentages are reported for the complete sample or as percentages (*n/N*) when the analyses contain missing data.

### Data Analysis

Univariate means or percentages were first calculated for sociodemographic variables in the entire sample and then by sex. Descriptive statistics were subsequently calculated to determine homogeneity between data collection points and sites (semesters and courses). When differences were negligible, data were pooled.

Categories for items regarding awareness of HPV and number of doses were collapsed into dichotomous outcomes. Likert questions were collapsed into “strongly agree/agree,” “disagree/strongly disagree,” and “neutral/don’t know.” Likert items were reverse scored, if necessary, to allow results to be analyzed together.

Correlates of five HPV-related outcomes were explored: awareness, self-perceived knowledge, measured knowledge, willingness to vaccinate, and vaccine beliefs. Sociodemographic characteristics, such as age, sex, race, ethnicity, health insurance status, HPV awareness, and HPV vaccine uptake, were among potential correlates.

Differences between men and women in binomial outcome variables were determined using Pearson’s chi-square or Fisher’s exact tests. General linear modeling was utilized to assess sex and age differences in participants’ mean knowledge score and the associations between perceived knowledge and knowledge score. Tukey’s post hoc test identified where significant differences occurred in age differences in the knowledge score. Mantel–Haenszel tests were conducted to determine sex differences in vaccine attitudes among vaccinated versus unvaccinated participants, and risk estimates to indicate the strength of the association were determined.

Binary logistic regression was used to evaluate demographic factors associated with binary vaccine initiation, HPV knowledge score, and HPV attitudes. The factors used to evaluate these outcomes were age, sex, ethnicity, and sexual orientation. A post hoc power analysis was conducted with GPower (Faul, Erdfleder, Lang, & Buchner, 2007), which indicated that a sample size of 358 was needed to detect effect sizes of *w* = 0.5 for tests of independence. Statistical significance was set at *p <* .05. All analyses were performed using SPSS 20.0 (IBM Corp., 2011).

## Results

### Sample Characteristics

Of the 409 students eligible to participate, 389 (95%) completed the survey and were included in the analysis. Three participants were under the age of 18 years and were excluded. Participants had a mean age of 22.3 years (*SD* = 4.9) and were predominantly female (70%). Approximately two thirds were Hispanic (66%) and single/unmarried (66%) and over half identified their race as White (59%). Men were more likely to report greater than 10 lifetime sexual partners than women were (28% vs. 10%, respectively; Table 1).

**Table 1.** Sociodemographic Characteristics of Participants by Gender.

	Total (*n* = 386)^a^	Female (*n* = 264)	Male (*n* = 110)
Characteristic	Mean (*SD*)	Mean (*SD*)	Mean (*SD*)
Age	22.4 (5.0)	22.2 (4.5)	22.6 (5.4)
Ethnicity	*N*	*n* (*%*)	*n* (*%*)
Hispanic/Latino	248	171 (63.6)	77 (67.5)
Not Hispanic/Latino	128	95 (35.3)	34 (29.8)
Not sure/unknown	7	3 (1.1)	3 (2.6)
Race/ethnicity			
White/Caucasian	235	164 (62.4)	75 (66.4)
Black/African American	81	57 (21.7)	25 (22.1)
More than one race	27	23 (7.6)	3 (2.7)
Not sure/unknown	17	12 (4.6)	5 (4.4)
Asian/Pacific Islander	14	9 (3.4)	5 (4.4)
American Indian/Alaska Native	2	1 (0.4)	0 (0)
Sexual orientation			
Straight	248	184 (68.1)	64 (57.1)
LGBT	134	86 (31.9)	48 (42.9)
Lifetime sexual partners			
0	54	38 (14.1)	16 (14)
1–5	214	166 (61.7)	48 (42.1)
6–10	57	39 (14.5)	18 (15.8)
>10	58	26 (9.7)	32 (28.1)
Health insurance			
Insured	238	161 (75.9)	77 (82.8)
Uninsured	53	42 (19.8)	11 (11.8)
Don’t know	14	9 (4.2)	5 (5.4)
Relationship status			
Single	254	171 (63.1)	83 (72.8)
In a relationship	111	85 (31.4)	26 (22.8)
Married	16	12 (4.4)	4 (3.5)
Divorced/widowed	4	3 (1.1)	1 (0.9)

*Note.* LGBT = lesbian, gay, bisexual, transgender.

a Includes 12 participants who did not specify their gender.

### HPV Awareness and Vaccination Status

Overall, the majority (84%) of participants had heard of HPV; more women were aware of the virus than men were (86% vs. 77%, respectively), although this difference was not significant (*p =* .07). The number of lifetime sex partners was not associated with HPV awareness; however, participants who reported being single (46.2%) were less likely to be aware of HPV than those in a relationship were (53.8%; *p =* .01). No significant differences were identified by race (e.g., Black, White, other), insurance status, or age.

Despite most participants’ being aware of HPV, the majority (60%) either had not received any HPV vaccine doses or did not know their vaccination status at the time of the survey. Participants who had received any doses of HPV vaccine were more likely to report having heard of HPV (*p* < .001; Table 2). Although 40% of respondents had at least one dose of HPV vaccine, only 15.3% had received three doses.

**Table 2.** Differences in HPV-Related Awareness, Perceived Knowledge, and Vaccine Attitudes by Vaccine Initiation.

Characteristic	*n*	0 Doses: HPV vaccine *n* (%)	≥1 Dose: HPV vaccine *n* (%)	*p* value
Heard of HPV before today				
Yes	257	143 (77.7)	114 (92.7)	.001
No/don’t know	50	41 (22.3)	9 (7.3)	
Perceived HPV knowledge				.007
Nothing/a little	216	140 (76.1)	76 (61.8)	
Moderate/a lot	91	44 (23.9)	47 (38.2)	
Measured HPV knowledge				<.001
Low score	196	133 (73.4)	63 (52.9)	
High score	104	48 (26.5)	56 (47.1)	
Vaccine attitudes				<.001
Positive	210	105 (57.7)	105 (88.2)	
Negative	91	77 (42.3)	14 (11.8)	

*Note. p* values for chi-square tests of vaccinated versus unvaccinated; all *p* values are significant. HPV = human papillomavirus.

Significant vaccination differences between men and women were evident. As shown in Table 3, the odds of women initiating HPV vaccination was over two times that of men, controlling for age, ethnicity, and sexual orientation (adjusted odds ratio [aOR] = 2.25, 95% confidence interval [CI] [1.27, 3.98], *p* = .005). Further, 72% of men had received no or an unknown number of doses of HPV vaccine compared to 55% of women (*p =* .01). Only 4% of men had all three doses as compared to 26% of women (*p* < .001). Finally, 45% of men and 27% of women said that they had never thought about getting the HPV vaccine for themselves (an additional 23% of men selected the option “don’t know”; *p <* .001).

**Table 3.** Correlates of HPV Vaccine Uptake, HPV Knowledge Score, and HPV Attitudes.

Variable	HPV vaccine uptake			HPV knowledge score			HPV attitudes		
	Odds ratio	95% CI	*p* value	Odds ratio	95% CI	*p* value	Odds ratio	95% CI	*p* value
Age	1.05	[0.99, 1.11]	.077	1.11	[1.04, 1.17]	**.001**	1.02	[0.97, 1.07]	–
Gender	2.26	[1.27, 3.98]	**.005**	1.05	[0.63, 1.76]	.830	1.36	[0.82, 2.25]	–
Ethnicity	1.09	[0.64, 1.83]	.750	0.67	[0.40, 1.13]	.134	1.23	[0.75, 2.01]	–
Sexual orientation	1.04	[0.60, 1.78]	.892	0.90	[0.55, 1.49]	.696	1.40	[0.84, 2.32]	–

*Note. p* values for binary logistic regression; *p* values in bold are significant. HPV = human papillomavirus.

### Perceived and Measured HPV-Related Knowledge

In response to the question “How much would you say you know about HPV?” (perceived knowledge), 69% of men and 62% of women perceived that they knew nothing or little and even more reported knowing nothing or little about the HPV vaccine (72% men and 67% women). Seventy percent of participants scored low (≤3 correct out of 7) on the true/false knowledge section. There were no significant differences between men and women in overall mean knowledge score; men earned an average score of 2.1 out of 7 (*SD* = 2.1) and women earned an average of 2.5 (*SD* = 1.9). Further, men (38%) were more likely than women were (24%) to score 0 points (*p =* .005). Only seven participants (1.8%) answered all seven questions correctly.

Age was significantly associated with knowledge score; the odds of older students receiving a high score was 10% higher than that of younger students (aOR = 1.11, 95% CI [1.04, 1.17], *p* = .001; Table 3). In addition, 82% of 18- to 20-year-olds in this sample were categorized as having low knowledge versus 62% of 21- to 23-year-olds (*p <* .001). A simple main-effects analysis showed that being in this age group did not have a statistically significant influence on men’s knowledge scores (*F*(1, 106) = 0.13, *p =* .72). Age was, however, a significant factor in women’s HPV knowledge score (*F*(3, 256) = 11.3, *p <* .001). Age was further analyzed in women by pairwise comparisons, using Tukey’s post hoc test, which showed that women in the age group of 18- to 20-year-olds (*n* = 99) had a significantly lower mean knowledge score than the other three age groups did (*n* = 166), and knowledge progressively increased with age (*p <*.001).

At least half of the respondents incorrectly identified herpes or AIDS as diseases caused by HPV (58% and 50%, respectively), with no significant differences by sex. Participants who received any doses of HPV vaccine (47%) or had >10 lifetime sexual partners (46%) were significantly more likely to score high on the knowledge items than others were (*p <* .001 and *p=* .02, respectively).

There was a strong relationship between perceived and actual HPV-related knowledge. Respondents who characterized their HPV-related knowledge as low (77%) earned low knowledge scores (*p <* .001). Only 51% of those with a high score perceived themselves to be knowledgeable. Similar to knowledge score and age, there was a significant difference between men and women. A simple main-effects analysis showed that men’s perceived knowledge was not independently associated with men’s knowledge scores (*F*(1, 109) = 2.6, *p =* .11). Among women, however, perceived knowledge was significantly associated with HPV knowledge score (*F*(1, 263) = 12.7, *p <* .001), with women who perceived themselves as knowing moderately/a lot about HPV performing better in knowledge score (*M* = 3.0, *SE* = 0.19) than those who perceived themselves to have no/little HPV knowledge did (*M* = 2.1, *SE* = 0.15, *p <* .001).

### Vaccine Attitudes

In response to the five-item vaccine attitudes scale, 70% of participants reported positive attitudes toward HPV vaccination, with men and women responding similarly. The number of doses of HPV vaccine received was significantly associated with vaccine attitudes, an association that differed between men and women (*χ*^2^_MH_(1) = 30.9, *p <* .001). Vaccinated women were over seven times more likely than unvaccinated women were to report positive vaccine attitudes (relative risk [RR] = 7.1, 95% CI [3.3, 15.5]). This stands in contrast to men, whose vaccination status was not significantly associated with vaccine attitudes (RR = 2.9, 95% CI [0.9, 8.7]).

Of the participants who reported negative attitudes toward vaccines, 85% had received either no or an unknown number of HPV vaccinations (*χ*^2^ = 31.8, *p <* .00). Eighty percent of participants with negative HPV vaccine attitudes scored low (<3/7) on the measured knowledge portion of the questionnaire (*χ*^2^ = 7.6, *p =* .01). Of those not vaccinated or undervaccinated (vaccine series initiated but incomplete), 58% were willing to receive the HPV vaccine and 27% did not know whether they wanted to be vaccinated. Participants who had heard of HPV before the day of the survey were more willing to be vaccinated than those who had not heard of the virus previously were (67% vs. 27%; *χ*^2^ = 34.8, *p <* .001).

## Discussion

Vaccinating both men and women against HPV is essential to protect against the rising HPV-related cancer rate. This analysis focused on differences between men and women in HPV awareness, HPV vaccine uptake, perceived and actual HPV knowledge, and vaccine attitudes. The men in this sample had scores that were close to those of women in awareness and knowledge, although women were still “ahead” in HPV vaccine uptake and vaccination intentions.

### Awareness

The male and female college students in this study had high levels of HPV awareness (>80%) compared to those in a 1999 study at a Florida university in which the students were largely oblivious to HPV; only 37% had ever heard of the virus (Yacobi, Tennant, Ferrante, Pal, & Roetzheim, 1999). The disparity between men and women may be closing as a result of an increase in overall awareness, with no significant differences by sex found in the present study. This is an apparent improvement compared with earlier research that found that college women were significantly more aware of HPV than college men were (Fontenot, Fantasia, Sutherland, et al., 2016; Gerend & Magloire, 2007; Osazuwa-Peters et al., 2017; Sandfort & Pleasant, 2009). Although awareness of the virus itself may be improving among men, we can continue this trend by ensuring that men are the recipients of detailed education efforts.

Kellogg et al. (2019) found that college men (65%) were significantly less aware of HPV vaccine age recommendations than college women were (51.6%). Misconceptions related to the appropriate age to get vaccinated may lead vaccine-eligible men to not initiate vaccination, despite being aware of HPV. Considering the recent changes in HPV vaccine recommendations, which increase the maximum age to initiate vaccination, rise in media coverage related to HPV vaccination may usher a surge in vaccination awareness.

### Perceived and Measured Knowledge

Despite high levels of HPV awareness among undergraduate students in South Florida, both men and women reported disconcertingly low levels of perceived and measured knowledge on the topic. Poor knowledge scores, especially among the youngest participants, who would have the most to benefit from vaccination, confirmed their self-perceptions. Of the men in this study, 38% answered no questions correctly on HPV knowledge. These results are consistent with previous studies that indicated that despite being acutely aware of HPV and HPV vaccination, significant knowledge deficits persist regarding how HPV is transmitted, what diseases result from HPV infection, and how to prevent infection effectively (Barnard, George, Perryman, & Wolff, 2017). Of various STIs evaluated in the late 1990s and early 2000s, college students knew the least about HPV (Bynum, Brandt, Friedman, Annang, & Tanner, 2011; Yacobi et al., 1999) and, as in this study, they confused HPV outcomes with herpes simplex virus or HIV (Garcini et al., 2015). A recent survey of college students found limited health literacy, with men scoring significantly higher than women (Albright & Allen, 2018). Despite differences in health literacy and similar HPV knowledge, college women were still more likely to be aware of HPV infection and more likely to be fully vaccinated (44% vs. 11%).

The youngest women received significantly lower scores than women in older age groups, similar to findings in previous research on age differences (Garcini et al., 2015). HPV knowledge disparities by age may be explained by older women having more encounters with sexual health education, undergoing HPV screening in the medical setting, knowing someone who has been diagnosed with an HPV infection or related disease, or experiencing infection themselves.

After the quadrivalent vaccine was introduced and approved for girls and women in 2006, there was intense marketing from the pharmaceutical manufacturers and health agencies (Fernandez, 2012). The manufacturer’s decision to market the vaccine to middle-class consumers as an “anticancer vaccine,” however, effectively detached cervical cancer from its sexually transmitted origins and increased attention on the vaccine’s acceptance by boys and men. While this approach may have reduced some of the stigma about a vaccine for an STI, a campaign that provided little HPV information may have increased awareness but failed to increase scientific knowledge (Rothman & Rothman, 2009). Low knowledge combined with high awareness may also reflect a lack of comprehensive sexual education during the formative years. For over 20 years, Florida public schools have adhered to an abstinence-only or abstinence-based sexual health curriculum (Barr, Moore, Johnson, Forrest, & Jordan, 2014). Despite this mandate, Florida lacks consistent state standards for the sexual education that is provided, with many students receiving no sexual health education at all. These results highlight the need for comprehensive sexual health education at all ages, whether it is received from a health-care provider, a school-based program, or another reliable source.

### Attitudes

This study explored attitudes toward HPV vaccination and how those attitudes may be associated with vaccination intentions and behaviors. In this study, over 70% of men had not been vaccinated and only 26% of women and 4% of men had received all three doses of HPV vaccine, despite men reporting similar awareness and knowledge as women. Participants reported favorable vaccine attitudes (70%), yet vaccinated women were seven times more likely to have positive views of vaccination than unvaccinated women were, a distinction not observed in young men. Taken together with the finding that most men had never thought about getting the vaccine for themselves, this suggests that men in this study who did initiate the HPV vaccine may not have received information about HPV or the HPV vaccine at the time of vaccination.

The first vaccines approved in 2006 were initially promoted for girls, creating earlier mindfulness of HPV and prevention among them. Previous studies with young men have reported that acceptability of the HPV vaccine has varied from modest (33%–48%) to high (78%; Brewer & Fazekas, 2007; Ferris et al., 2009; Lenselink et al., 2008; Reiter, Brewer, & Smith, 2010). While vaccine hesitancy was not addressed in this study, its influence may be responsible for the attitudinal differences between those who chose to initiate the HPV vaccine series and those who did not. Depending on age at vaccination, this hesitance is likely derived from the parents accompanying their children on their vaccination visit rather than sentiments of the patients themselves. The case also has been made for an altruistic motivation for men to be vaccinated, thereby protecting women through their vaccination (Bonafide & Vanable, 2015). The steep rise in HPV-related oropharyngeal cancer morbidity in men and the potential protective effect of the HPV vaccine for oropharyngeal infection suggest an urgent need to improve vaccine uptake in men to protect themselves from adverse health consequences (Chaturvedi, Engels, Anderson, & Gillison, 2008; Chaturvedi et al., 2018).

Those with negative attitudes toward HPV vaccination were overwhelmingly uninformed about HPV and were likely to be unvaccinated. This finding parallels recent research that describes HPV knowledge as a predictor of attitudes toward vaccination (Otanez & Torr, 2018). The positive correlation between a favorable attitude and vaccine uptake may be an effect of attitude–behavior consistency. Longitudinal attitude studies have reported that positive correlations between attitude and behavior strengthen as a function of information available on the topic as well as direct experience with the attitude object (Davidson, Yantis, Norwood, & Montano, 1985; Fabrigar, Petty, Smith, & Crites, 2006). In this sample, those with positive attitudes tended to be more knowledgeable but also were more likely to already have received at least one dose of HPV vaccine.

### Limitations

The limitations of this study include its cross-sectional nature, which precludes the ability to infer causation or to establish a temporal connection in variables of interest, such as HPV knowledge and vaccine uptake. Self-reported data were collected from the participants, which may subject our results to social desirability and recall biases. The possible effects of social desirability bias, which may decrease the validity of the reported information, are estimated to be minimal in this study. The survey was an anonymous, self-administered questionnaire, which is likely to reduce the respondents’ desire to present themselves in a favorable manner (Gonyea & Umbach, 2005).

Self-reported data also can be compromised by the respondents’ memory of events or experiences of interest. Recall bias often can decrease the reliability of information collected (Gonyea & Umbach, 2005). Existing studies suggest that self-reported HPV vaccine initiation can be a valid and reliable measure among young adults (Grimaldi-Bensouda et al., 2013; Niccolai, McBride, Julian, & the Connecticut HPV-IMPACT Working Group, 2014; Rolnick et al., 2013). Approximately 93% of men 22 to 26 years old self-reported accurate vaccination (Thomas et al., 2018). The use of “don’t know” as a response option for vaccination status in the current study may have contributed to further precision of the responses.

These results should be interpreted within the context of the timing of data collection for this survey. The average age of men in this study was 22.6 years. Young men attending college from 2015 to 2017, when this survey was conducted, were most likely already in their mid- to late teens when the ACIP recommendation for males was issued in 2011. Opportunities to receive catch-up vaccination against HPV until age 21 years (or until age 26 for men considered high risk) may have been few for male respondents of this survey. Additional changes to the recommended adult immunization schedule have recently occurred to reflect the Food and Drug Administration’s recent approval of the HPV vaccine for adults up to age 45 years (U.S. Food and Drug Administration, 2018). The ACIP now recommends catch-up HPV vaccination until age 26 years for those not adequately vaccinated and recommends vaccination based on a shared decision with health-care providers for those aged 27 through 45 years who are not adequately vaccinated (Meites et al., 2019).

This study used a nonprobability sample. Recruitment was conducted in an urban area of south Florida, a state among those with the highest percentages of Hispanic and foreign-born residents (Pinheiro et al., 2009). Florida, specifically Miami-Dade County, leads the country in number of Cuban-descent residents, who are typically not representative of Hispanics from other countries. Despite being a middle-income country, Cuba performs well in national health indicators when compared to other countries and is ranked higher than the United States, according to the Bloomberg Healthiest Country Index (2019). Aside from demonstrating success in universal health coverage and access (World Health Organization, 2015), Cuba has developed and applied innovative biotechnological approaches to mitigating cervical cancer (Gonzalez-Fernandez & Gonzalez-Fernandez, 2016). As residents of Cuban descent acculturate in the United States with varying levels of transnationalism, it is likely that they bring these health practices with them, which may be reflected in the outcomes presented here.

Despite these limitations, these results provide evidence that a significant number of young adults in the United States lack accurate knowledge about HPV, the HPV vaccine, and the importance of preventing HPV infection.

### Implications and Future Research

The results of this analysis suggest that educational interventions should be delivered to increase knowledge and change attitudes toward HPV and HPV vaccination among college students, especially men and freshman women. The college setting provides an optimal environment for education about the virus and uptake of the vaccine. Further, student health centers can assist in removing structural barriers that prevent vaccination among those who are knowledgeable (Dillard & Spear, 2006; Fontenot, Fantasia, Vetters, & Zimet, 2016).

Future research can integrate these findings to support research aimed at improving knowledge and attitudes regarding HPV among men. Qualitative research can be conducted with at-risk groups, such as men, to inform the design and development of tailored interventions. This would be useful in determining what messages are suitable for this population as well as who provides messages, where messages are delivered, and which approaches should be utilized (Catalano et al., 2017).

To expedite the achievement of state and national vaccination goals, several actions can be pursued at the state level. Florida and other states can review options for the implementation and evaluation of comprehensive sexual education for elementary, middle, and high school students. Many studies have reported support for such programs from both teachers and parents (Barr et al., 2014; Howard-Barr, Moore, Weiss, & Jobli, 2011). Because there is no cure for HPV or other viral STIs, primary prevention is essential. Florida and other states could follow the lead of states such as Virginia, Rhode Island, and the District of Columbia in requiring HPV vaccination, along with other routine vaccinations, for school matriculation (National Conference of State Legislatures, 2017). States that have standard comprehensive sexual education as well as mandated HPV vaccination were found to have higher HPV vaccine completion rates (Franco, Mazzucca, Padek, & Brownson, 2019).

In 2012, Senate Bill 146, which proposed that parents and caregivers of students who are entering sixth grade be given information about HPV, related diseases, and the availability of vaccines to prevent transmission, was introduced in the Florida Senate (Altman, 2013). Unfortunately, the bill died in the Health Policy Committee. In January 2018, Senate Bill 1558, which would make it mandatory for 11- and 12-year-olds who attend public school to become vaccinated against HPV, was introduced in Florida (Rodriguez, 2018); this bill also died in the Health Policy Committee.

If HPV vaccination also were required for college matriculation, dramatic reductions in HPV-related outcomes might be seen. Increased gender-inclusive, targeted information for college students in college clinical settings would reinforce health promotion messages. To remove barriers such as cost and access, college health centers and clinics could determine mechanisms to offer HPV vaccination to students free of charge. The vast majority of the students in this study had health insurance coverage, which includes HPV vaccination, at the time of data collection. The institution at which this study was carried out houses a full-service health center that offers HPV vaccination and also participates in programs designed to reduce monetary costs. Pharmaceutical companies often offer vouchers to institutions such as qualified colleges and universities to, in most cases, completely subsidize such costs, which could cover the costs for uninsured students. Since college health services may be limited in ways they can support undervaccinated students, building capacity for college health centers to expand primary care services and integrate with state immunization registries should be prioritized (Lemly, Lawlor, Scherer, Keleman, & Weitzman, 2014).

At doctor visits off campus, providers could conduct a vaccine eligibility assessment, regardless of sex, at each visit and during general non-STI visits. National vaccine registries and reporting systems should be integrated and intuitive for clinicians, which would help to strengthen vaccine reporting and support vaccine recommendation equality.

## Conclusions

The majority of the HPV burden is carried among 18- to 26-year-olds across the United States and there is a distinct opportunity for preventive measures to be improved among this group. The findings of this study indicate the need for aggressive targeting of young adults for educational and behavioral interventions, with particular attention to increasing knowledge among both men and women. Despite general awareness of *HPV* and *HPV vaccine* as hot-button terms, participants in this study demonstrated a lack of detailed knowledge about HPV, which, in turn, is associated with negative vaccine attitudes and uptake. Knowledge among young men seems to be slowly improving, yet continued vaccination deficits place an unreasonable burden on women to protect against a virus that increasingly affects men. Improvements in young adults’ awareness, knowledge, and attitudes toward HPV and HPV vaccination are imperative to reach the goal of 80% HPV vaccination coverage to reduce the burden of HPV-related disease in the United States.

## Appendix

**Appendix 1.** Knowledge Items from the Questionnaire.

Item	Correct answer
Q1. HPV infection is rare	False
Q2. HPV causes herpes	False
Q3. AIDS is caused by HPV	False
Q4. The only way to protect yourself from HPV is to use a condom	False
Q5. Some strains of HPV are harmless	True
Q6. HPV infection can cause cancer	True
Q7. Student Health Services offers HPV vaccination	True
